# INTERROGATING THE SKILLED NURSING STAYS OF HOMELESS AND SERIOUSLY ILL OLDER ADULTS

**DOI:** 10.1093/geroni/igad104.1194

**Published:** 2023-12-21

**Authors:** Namrata Mukherjee, Ian Johnson, Michael Light

**Affiliations:** University of Tennessee, Knoxville, Knoxville, Tennessee, United States; University of Tennessee, Knoxville, Tennessee, United States; University of Washington Palliative Care Training Center, Seattle, Washington, United States

## Abstract

The culture change movement has fostered an intentional move away from restrictive ethos of care in hospitals, post-acute rehabilitation, and skilled nursing. However, for the millions in the United States who are uninsured, Medicaid-insured, or dual-eligible are less likely to access ‘deinstitutionalized’ healthcare environments. This presentation presents findings from a constructivist grounded theory study, Research and Supportive Care at Later-life for Unhoused People (RASCAL-UP), on the role of skilled nursing in the experiences of palliative care patients with histories of homelessness. Through provider interviews (n=30), field observations (n=12), and longitudinal chart documentation of a subset of homeless palliative care patients with stays in skilled nursing (n=13), this presentation will reflect on major findings: (1) barriers to skilled nursing entry; (2) successes and challenges in transitioning into skilled nursing residence; (3) acts of resistance to (re)institutionalization; and (4) discharge options and outcomes. Results showcase a need to fill the residential service gap between supportive housing and skilled nursing care and policy change that incentivizes consumer-directed practices for all.

